# Effects of different sufentanil target concentrations on the MAC_BAR_ of sevoflurane in patients with carbon dioxide pneumoperitoneum stimulus

**DOI:** 10.1186/s12871-020-01160-1

**Published:** 2020-09-21

**Authors:** Yanxia Guo, Dan Wang, Xiaolin Yang, Pingping Jiang, Juan Xu, Guoyuan Zhang

**Affiliations:** 1 Department of Anaesthesiology, Affiliated Hospital of North Sichuan Medical College, Nanchong, 637000 Sichuan China; 2 Department of Clinical Laboratory, Affiliated Hospital of North Sichuan Medical College, Nanchong, 637000 Sichuan China

**Keywords:** Anesthetics, inhalation, Sufentanil, Pneumoperitoneum

## Abstract

**Background:**

This study aims to observe the effects of different target controlled plasma sufentanil concentrations on the minimum alveolar concentration (MAC) of sevoflurane for blocking adrenergic response (BAR) in patients undergoing laparoscopic cholecystectomy with carbon dioxide pneumoperitoneum stimulation.

**Methods:**

Eighty-five patients undergoing laparoscopic cholecystectomy, aged 30–65 years, with American Society of Anesthesiologists physical status I-II, were enrolled in this study. All the patients were randomly divided into 5 groups (S_0_, S_1_, S_2_, S_3_, S_4_) with different sufentanil plasma target concentration (0.0, 0.1, 0.3, 0.5, 0.7 ng ml^− 1^). Anesthesia was induced by inhalation of 8% sevoflurane in 100% oxygen, and 0.6 mg kg^− 1^ of rocuronium was intravenously injected to facilitate the insertion of a laryngeal mask airway. The end-tidal sevoflurane concentration and sufentanil plasma target concentration were adjusted according to respective preset value in each group. The hemodynamic response to pneumoperitoneum stimulus was observed after the end-tidal sevoflurane concentration had been maintained stable at least for 15 min. The MAC_BAR_ of sevoflurane was measured by a sequential method. Meanwhile, epinephrine (E) and norepinephrine (NE) concentrations in the blood were also determined before and after pneumoperitoneum stimulus in each group.

**Results:**

When the method of independent paired reversals was used, the MAC_BAR_ of sevoflurane in groups S_0_, S_1_, S_2_, S_3_, S_4_ was 5.333% (confidence interval [CI] 95%: 5.197–5.469%), 4.533% (95% CI: 4.451–4.616%), 2.861% (95% CI: 2.752–2.981%), 2.233% (95% CI: 2.142–2.324%) and 2.139% (95% CI: 2.057–2.219%), respectively. Meanwhile, when the isotonic regression analysis was used, the MAC_BAR_ of sevoflurane in groups S_0_, S_1_, S_2_, S_3_, S_4_ was 5.329% (95% CI: 5.321–5.343%), 4.557% (95% CI: 4.552–4.568%), 2.900% (95% CI: 2.894–2.911%), 2.216% (95% CI: 2.173–2.223%) and 2.171% (95% CI: 2.165–2.183%), respectively. The MAC_BAR_ was not significantly different between groups S_3_ and S_4_ when using 0.5 and 0.7 ng ml^− 1^ of sufentanil plasma target concentrations. No significant difference was found in the change of E or NE concentration between before and after pneumoperitoneum stimulation in each group.

**Conclusions:**

The MAC_BAR_ of sevoflurane can be decreased with increasing sufentanil plasma target concentrations. A ceiling effect of the decrease occurred at a sufentanil plasma target concentration of 0.5 ng ml^− 1^. When the sympathetic adrenergic response was inhibited in half of the patients to pneumoperitoneum stimulation in each group, the changes of E and NE concentrations showed no significant differences.

**Trial registration:**

The study was registered at http://www.chictr.org.cn (ChiCTR1800015819, 23, April, 2018).

## Background

With the development of minimally invasive techniques, laparoscopic surgery under inhalation anaesthesia has become increasing popular in general surgery [1–3]. However, inhalation anaesthetic used alone to provide all the necessary components of general anesthesia under laparoscopic surgery may increase the risk of cardiovascular inhibition and inhaled anaesthetic toxicity [4–6]. Many agents have been used to decrease the minimal alveolar concentration (MAC) of inhalation anaesthetics [7, 8]. Sufentanil, as an adjuvant, offers numerous advantages, including a reduced incidence of postoperative nausea and vomiting compared with the fentanyl [9], reduced opioid-induced hyperalgesia compared with the remifentanil [10], maintenance of stable hemodynamics, excellent analgesic effect. The MAC of sevoflurane for blocking the adrenergic response (BAR) at different sufentanil plasma target concentrations under laparoscopic pneumoperitoneum stimulus has not been reported. Therefore, our primary aim of this study is to observe the MAC_BAR_ of sevoflurane combined with different sufentanil plasma target concentrations in patients under carbon dioxide pneumoperitoneum stimulation. A secondary aim is to explore the concentrations of epinephrine and norepinephrine in the blood when the adrenergic response was inhibited in half of the patients.

## Methods

### Study design

The study was approved by the Ethics Committee of Affiliated Hospital of North Sichuan Medical College, Nanchong, China (Approved No. 2017/043). Written informed consents were obtained from all participants. All experiment procedures (blood collections and arterial catheterization) and data collection were conducted with prior informed consents. This study adhered to the applicable CONSORT guidelines and was registered with the Chinese Clinical Trials Registry at http://www.chictr.org.cn (ChiCTR1800015819, principal investigator: Yanxia Guo, date of registration: April 23, 2018).

The research was conducted between May 2018 and March 2019. Eighty five American Society of Anesthesiologists (ASA) physical status I-II, patients aged between 30 and 65 years, were randomly assigned to five groups (S_0_, S_1_, S_2_, S_3_, S_4_) according to a computer generated randomization. Patients in the five groups were anaesthetized by mask inhalation of sevoflurane and intravenous infusion of sufentanil with different plasma target concentrations: 0.0, 0.1, 0.3, 0.5, 0.7 ng ml^− 1^. Exclusion criteria included that: patients with a history of cardiovascular, lung, kidney or brain disease; long-term drug or alcohol abuse; recent take drugs known to affect the sympathetic adrenergic and cardiovascular systems; and body mass index (BMI) ≧30 kg m^− 2^. Withdrawal criteria included patients with mean arterial pressure (MAP) < 50 mmHg or heart rate (HR) < 50 bpm at any time during experimental observation; failing to achieve creation of the carbon dioxide pneumoperitoneum on the first attempt; or asking for adjustment of the pneumoperitoneal pressure above or below the preset value.

### Anaesthesia administration

#### Induction

All patients were fasted at least for 8 h before surgery and without any preoperative medication. Before induction of anaesthesia, patient’s MAP, HR, electrocardiogram, and oxygen saturation were monitored as per routine with a PM-9000 express monitor (Mindray Medical International Limited, Shenzhen, China). Simultaneously, a peripheral intravenous catheter was inserted for infusion of Ringer’s solution at a rate of 10 ml kg^− 1^ h^− 1^. An arterial catheter was inserted into the left radial artery for monitoring patient’s arterial blood pressure and collecting blood samples. Anaesthesia was induced by inhalation of 8% sevoflurane with 100% oxygen until patients lost their consciousness, then 0.6 mg kg^− 1^ of rocuronium was intravenously injected to facilitate the insertion of laryngeal mask airway (Tuoren medical equipment group co. LTD, Henan, China) insertion. Then mechanical ventilation was begun using 100% oxygen with a tidal volume of 6 to 8 ml kg^− 1^. A normal end tidal carbon dioxide (CO_2_) tension (35 to 45 mmHg) was obtained by adjusting the respiratory frequency at 12 to 16 breaths min^− 1^. The end-tidal sevoflurane concentration and CO_2_ partial pressure were monitored continuously using the above-mentioned monitor. Depth of anaesthesia was monitored by the bispectral index (BIS) (Canwell Medical International Limited, Zejiang, China) which was placed before induction.

#### Measurement of MAC_BAR_

After laryngeal mask airway insertion, sufentanil was administered by target-controlled infusion with Bovil pharmacokinetic model using a micro pump (TCI-I, ver 4.0, Guangxi VERYARK Technology Co., Ltd), and the plasma target concentration of sufentanil was 0.0, 0.1, 0.3, 0.5, 0.7 ng ml^− 1^ in groups S_0_, S_1_, S_2_, S_3_, S_4_, respectively. Simultaneously, the inhaled sevoflurane concentration was adjusted to obtain a stable preset end-tidal value according to our pilot study. In order to avoid a potential risk of intraoperative awareness, a higher initial end-tidal sevoflurane concentration was tested in the pilot study. The first patient in groups S_0_, S_1_, S_2_, S_3_ and S_4_ received a start end-tidal sevoflurane preset concentration of 5.0, 4.6, 3.0, 2.3 and 2.0% which was determined to be close to the MAC_BAR_, respectively. An up-and-down sequential-allocation method was applied to determine the MAC_BAR_ of sevoflurane in each group as described in our previous studies [11, 12].

The CO_2_ pneumoperitoneum was created when the preset end-tidal sevoflurane concentration had been maintained stable at least 15 min. The creation of pneumoperitoneum was initiated using a Veress needle with the CO_2_ pressure set to 13 mmHg at umbilicus and the insufflation flow rate was set at 3 L/min. After the CO_2_ pneumoperitoneum had been created a 10-mm trocar replaced the Veress needle. Another 10-mm trocar and a 5-mm trocar were installed through a subxiphoid port and a port in the right subcostal area of the midclavicular line, respectively. HR and MAP were determined before induction, 3 and 1 min before CO_2_ pneumoperitoneum, and 1 and 3 min after three trocars were installed. Presence or absence of a sympathetic adrenergic response during the creation of the CO_2_ pneumoperitoneum was indicated by HR or MAP was recorded. Both the mean value of MAP and the mean value of HR measured 3 and 1 min before pneumoperitoneum stimulation were defined as the pre-pneumoperitoneum values, and the mean value of HR and the mean value of MAP measured 1 and 3 min after the trocars had been installed were defined as the post-pneumoperitoneum values. If the response was positive (an increase of patient’s HR or MAP > 20% of its pre-pneumoperitoneum value), the subsequent tested patient’s end-tidal sevoflurane concentration would be increased by 0.2%. If the response was absent i.e. HR and MAP change of < 20% of its pre-pneumoperitoneum value, the subsequent tested patient’s end-tidal sevoflurane concentration would be decreased by 0.2%. Patients with bradycardia (HR < 50 bpm) or hypotension (MAP < 50 mmHg) at any time during experimental observation were administered vascular active drugs such as atropine, ephedrine, and withdrawn from the study, a same tested end-tidal sevoflurane concentration was repeated in the following case. The study was continued until six crossing points of a negative versus positive response in the pre-and the next patient had occurred. The investigator responsible for recording the response of the patients to CO_2_ pneumoperitoneum was blinded to the plasma target controlled sufentanil concentrations and end-tidal sevoflurane concentration used in all the study patients. The MAC_BAR_ of sevoflurane in each group was calculated as the mean value of the end-tidal sevoflurane concentrations corresponding to the six crossing points.

After the above test was completed, the target controlled infusion of sufentanil was stopped in each group. The patients in group S_0_ received an i.v. bolus of 0.3 μg kg^− 1^ sufentanil. Furthermore, the inspired concentration of sevoflurane was adjusted to maintain the end-tidal concentrations at 1.4–1.7 MAC for maintaining the BIS value between 40 and 60. MAP was maintained between 60 and 85 mmHg intraoperatively. If the MAP increased by more than 20% compared with its preoperative value, a bolus of 10 μg sufentanil was administered. After surgery and removal of the laryngeal mask airway, patients were transported to the post- anaesthesia care unit (PACU). In the PACU, all patients were asked about whether there was any intraoperative awareness or not.

#### Analysis of blood samples

Arterial blood samples were collected 3 min before and after CO_2_ pneumoperitoneum and stored in sodium-heparin-containing tubes. Soon after, the plasma was separated and kept frozen at − 70 °C until analysis. The method used to measure the concentrations of E and NE in the current investigation have been described previously [12].

### Statistical analysis

Statistical analysis was performed using SPSS 23.0 software. The MAC_BAR_ was estimated from the up-and-down sequences using the method of independent paired reversals, which enabled MAC_BAR_ with 95% CIs to be derived [13]. The sequences were also subjected to isotonic regression analyses. To compare the MAC_BAR_ from different groups more precisely, the 83% CIs were estimated using the isotonic regression analysis. The delta HR, delta MAP, delta E, delta NE value were calculated as the differences between their average values measured 1 and 3 min before and after CO_2_ pneumoperitoneum. The data are presented as mean (SDs or 95%CI). The preoperative data, including gender and ASA class were compared with X^2^ test. The preoperative data (age, BMI), the intraoperative data, the postoperative data, the MAC_BARS_, the concentrations of E and NE, delta E, delta NE, MAP, delta MAP, HR, and delta HR were compared among the 5 groups using one-way analysis of variance (ANOVA). *P* value < 0.05 was considered as statistical significance.

## Results

**Fig. 1:**
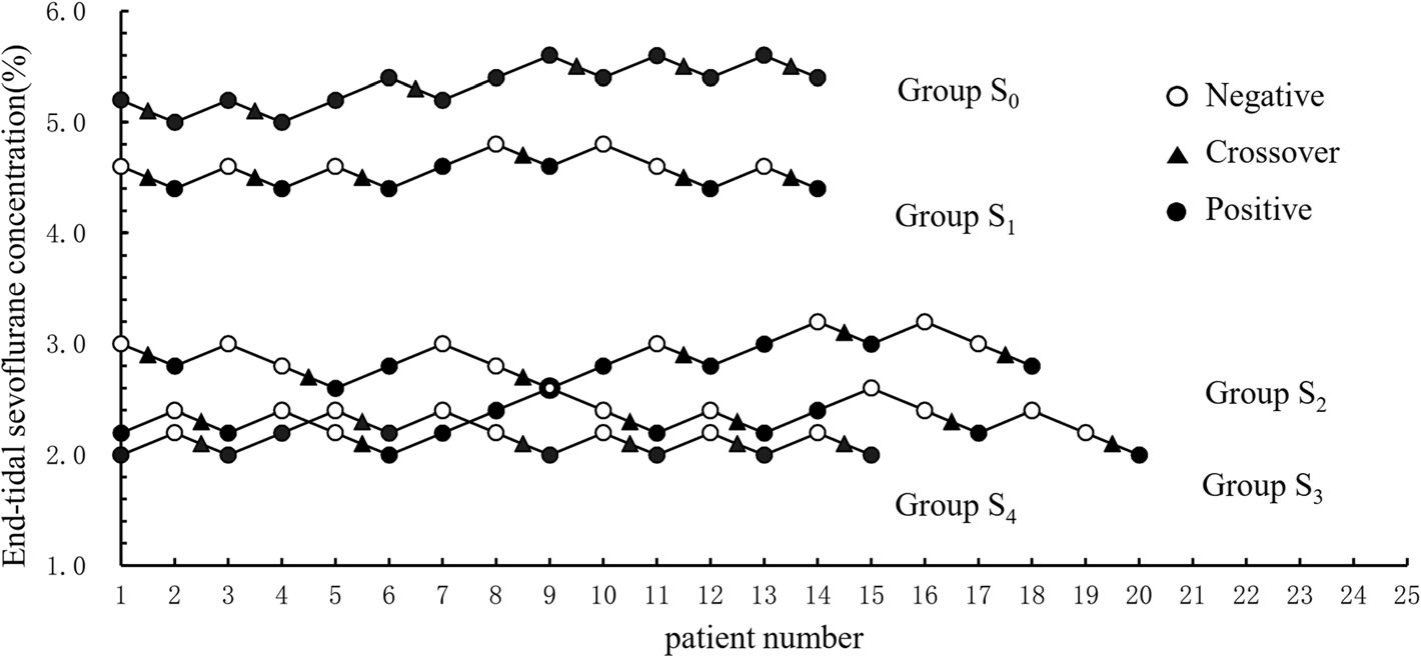
Dixon up-and-down plots for each group. The plasma target concentration of sufentanil in groups S_0_, S_1_, S_2_, S_3_ and S_4_ was 0.0, 0.1, 0.3, 0.5 and 0.7 ng ml^− 1^, respectively. The empty (solid) circle represents the negative (positive) reaction to hemodynamics parameters, and the triangle indicates the intersection of negative and positive reactions. The ninth patient was given the same concentration of sevoflurane both in group S_2_ and group S_3_. To get six crossovers, 14, 14, 18, 20 and 15 patients were needed in groups S_0_-S_4_, respectively

A total of 85 patients were recruited in this study. One case in group S_0_ and one case in group S_3_ both with MAP < 50 mmHg were removed from the study. Two cases with HR < 50 bpm in group S_4_ were also removed from the study. Ultimately, to obtain six crossing points, 14, 14, 18, 20 and 15 patients were used in groups S_0_-S_4_, respectively (Fig. 1), so that 81 patients completed the study. No significant differences were found in the patients’ preoperative data, operation time and rocuronium consumed among the 5 groups (Table 1). No intraoperative awareness was reported in the postoperative follow up.

**Table 1:** Patients characteristics and Intraoperative and Postoperative data

Parameter	Group S_0_	Group S_1_	Group S_2_	Group S_3_	Group S_4_
Preoperative data					
Gender (n, M/F)	6/8	6/8	6/12	10/10	7/8
ASA class (I/II)	7/7	6/8	10/8	10/10	8/7
Age (yr)	41 (8)	38 (9)	37 (10)	41 (11)	39 (9)
Body weight (kg)	68.3 (9.8)	65.7 (8.4)	67.2 (7.9)	66.2 (8.5)	67.3 (9.2)
BMI (kg m^− 2^)	23.1 (2.2)	23.4 (2.3)	23.1 (2.7)	23.6 (1.9)	24.0 (2.3)
MAP (mmHg)	92.8 (9.6)	89.8 (6.9)	91.2 (6.7)	89.2 (8.2)	89.9 (9.6)
HR (bpm)	82 (10)	77 (12)	82 (10)	83 (9)	78 (12)
Intraoperative data					
Operation time (min)	62.3 (7.9)	59.6 (8.1)	60.7 (8.9)	56.5 (9.2)	58.9 (5.0)
Total sufentanil consumed dose (μg)	31.4 (5.6)	29.8 (3.9)	30.3 (4.5)	44.6 (5.8)^*^	61.4 (4.8)^*#^
Rocuronium consumed dose (mg)	35.0 (5.0)	37.5 (6.3)	36.7 (4.9)	38.3 (4.6)	38.6 (6.2)
Postoperative data					
Spontaneous breathing recovery time (min)	5.2 (2.1)	4.8 (2.5)	5.0 (1.9)	4.5 (2.8)	10.0 (3.9)^#^
Eye opening time (min)	7.8 (3.1)	8.0 (1.9)	7.5 (1.8)	8.2 (3.3)	16.4 (5.2)^#^
Extubation time (min)	10.2 (1.7)	11.7 (2.3)	10.5 (1.7)	11.0 (3.0)	20.2 (3.8)^#^

Data are presented as mean (SD)

*P < 0.05 vs. the value of group S_0_, S_1_, S_2_, respectively. ^#^*P* < 0.05 vs. the value of S_0_, S_1_, S_2_, S_3_, respectively

The estimates of MAC_BAR_ of sevoflurane by the method of independent paired reversals and isotonic regression using the different plasma target concentration of sufentanil in groups S_0_-S_4_ are shown in Table 2. The 83% CIs were overlapped in group S_3_ and S_4_ using the isotonic regression analysis. For both methods, the MAC_BAR_ was not significantly different between group S_3_ and group S_4_ when using 0.5 and 0.7 ng ml^− 1^ of sufentanil plasma target concentrations. The HR and delta HR were similar among groups S_2_, S_3_, and S_4_, but significantly lower than groups S_0_ and S_1_ (*P* < 0.05, Table 3). No significant differences were found in the MAP, delta MAP, epinephrine and norepinephrine concentration, delta epinephrine and norepinephrine concentration among the 5 groups (Table 3). The total administered dose of sufentanil in both group S_3_ and group S_4_ was higher than in groups S_0_, S_1_, S_2_ (*P* < 0.05, Table 1). The spontaneous breathing recovery time, eye opening time and extubation time in group S_4_ was longer than those in the other 4 groups (*P* < 0.05, Table 1).

**Table 2:** The MAC_BAR_ of sevoflurane using the method of independent paired reversals and isotonic regression analyses in 5 groups

Group	Target concentration of sufentanil (ng ml^−1^)	Empirical mean MAC_BAR_ (95% CI)	Isotonic regression MAC_BAR_(95% CI), (83% CI)
S_0_	0.0	5.333 (5.197–5.469)	5.329 (5.321–5.343) (5.324–5.339)
S_1_	0.1	4.533 (4.451–4.616)^*^	4.557 (4.552–4.568) (4.555–4.566)
S_2_	0.3	2.861 (2.752–2.981)^*#^	2.900 (2.894–2.911) (2.898–2.909)
S_3_	0.5	2.233 (2.142–2.324)^*#※^	2.216 (2.173–2.223) (2.177–2.212)
S_4_	0.7	2.139 (2.057–2.219)^*#※^	2.171 (2.165–2.183) (2.170–2.180)

The data of MAC_BAR_ were presented as means (95% CI or 83% CI)

^*^P < 0.05 vs. value of group S_0_. ^#^*P* < 0.05 vs. value of group S_1_.^※^*P* < 0.05 vs. value of group S_2_

**Table 3:** The comparison of MAP, HR, epinephrine and norepinephrine concentrations before and after pneumoperitoneum stimulus among 5 groups

	Group S_0_	Group S_1_	Group S_2_	Group S_3_	Group S_4_
MAP (mmHg)					
Before pneumoperitoneum	63 (5)	62 (4)	64 (4)	63 (5)	62 (5)
After pneumoperitoneum	78 (8)	75 (8)	79 (8)	78 (7)	76 (6)
Delta	15 (7)	13 (8)	15 (6)	16 (2)	14 (8)
HR (bpm)					
Before pneumoperitoneum	89 (11)	82 (15)	67 (6)^*#^	61 (5)^*#^	62 (6)^*#^
After pneumoperitoneum	100 (13)	92 (15)	69 (7)^*#^	64 (8)^*#^	66 (6)^*#^
Delta	11 (5)	10 (4)	2 (1)^*#^	3 (1)^*#^	3 (2)^*#^
Epinephrine (ng ml^−1^)					
Before pneumoperitoneum	2.85 (0.23)	2.97 (0.19)	2.92 (0.19)	2.82 (0.28)	2.67 (0.18)
After pneumoperitoneum	2.92 (0.25)	3.04 (0.40)	2.91 (0.17)	2.85 (0.29)	2.62 (0.11)
Delta	0.07 (0.04)	0.07 (0.02)	0.03 (0.02)	0.03 (0.01)	−0.04 (0.03)
Norepinephrine (ng ml^− 1^)					
Before pneumoperitoneum	3.23 (0.21)	3.63 (0.23)	2.89 (0.19)	3.18 (0.95)	3.12 (0.74)
After pneumoperitoneum	3.11 (0.33)	3.55 (0.13)	2.81 (0.25)	3.15 (0.65)	3.07 (0.45)
Delta	−0.12 (0.07)	− 0.08 (0.04)	− 0.08 (0.05)	−0.03 (0.02)	− 0.05 (0.03)

The value of each parameter before pneumoperitoneum was the average value measured 3 and 1 min before CO_2_ pneumoperitoneum. The value of each parameter after pneumoperitoneum was the average value measured 3 and 1 min after CO_2_ pneumoperitoneum and the delta value of each parameter was the difference between the average value measured 1 and 3 min after CO_2_ pneumoperitoneum and before CO_2_ pneumoperitoneum value

^*^P < 0.05 vs. values of group S_0_. ^#^*P* < 0.05 vs. values of group S_1_

## Discussion

The results of this study indicate that the reduction of the MAC_BAR_ of sevoflurane by sufentanil is dose-dependent. The overlapped 83% CIs in group S_3_ and S_4_ using the isotonic regression analysis indicate the MAC_BAR_ had no significant difference when using 0.5 and 0.7 ng ml^− 1^ of sufentanil plasma target concentrations. This suggests that a ceiling effect of the decrease of MAC_BAR_ of sevoflurane occurred when the sufentanil plasma target concentration increased to > 0.5 ng ml^− 1^ (Table 2). This ceiling effect of sufentanil is similar to the result measured by Brunner and colleagues [14] at the same plasma target concentration when they evaluated the reduction of isoflurane’s MAC by sufentanil in response to skin incision. Sufentanil is a μ receptor agonist, which can be saturated when its plasma target concentration is beyond a certain level [15]. We speculate that a similar ceiling effect will occur under a similar plasma target concentration of sufentanil no matter what kind of surgery or stimulus is selected. The ceiling plasma concentration of sufentanil (0.18 ng ml^− 1^) in Shun-Huang and colleague’s study [16] is significantly lower than that of our experiment result. We believe that is reasonably explained by the concomitant administration of 60% nitrous oxide [17, 18]. Several studies show that nitrous oxide can combine with the μ receptor and decrease the available binding sites of sufentanil in humans [19–23].

In this study, the MAC_BAR_ of sevoflurane (5.333%) under laparoscopic pneumoperitoneum stimulation is higher than that measured by Katoh and his colleagues (4.15%) under skin incision [24]. It suggests that the laparoscopic pneumoperitoneum stimulus is stronger than the skin incision stimulus, so that a higher concentration of sevoflurane is needed to inhibit the stress reaction in laparoscopic surgery, which is consistent with the results of our previous study [25]. The MAC_BAR_ of sevoflurane measured in this study is also significantly higher than the value (4.6%) reported in our previous study in gynecologic patients [12]. Although the same CO_2_ pneumoperitoneum stimulus was used, the MAC_BAR_ of sevoflurane could also be affected by the location of the perforation for establishing pneumoperitoneum, the patient’s age and gender [26, 27], the methods of measurement [25, 26] and the criterion of judgment for a positive or negative response [27, 28]. Dixon thought that the MAC_BAR_ values could be estimated as the mean of four independent crossovers of responses [28]. Paul and his colleagues thought that the reliability of the Dixon method increased with the number of pairs and six pairs was enough [29]. An increase of 15% or more from the baseline value of MAP or HR was taken as the criterion of a positive response in many studies [8, 30]. However, in clinic, the fluctuation of MAP or HR within the range of 20% is also acceptable and reasonable. Therefore, in our current study, an increase of 20% or more from pre-pneumoperitoneum stimulation values of MAP or HR was taken as the standard to judge a positive response.

Our results indicated the delta E or NE concentrations did not differ among all 5 groups (Table 3). This observation implies that when the sympathetic adrenergic response was inhibited in half patients to pneumoperitoneum stimulation in each group, the change of E or NE concentration would be similar, no matter the target controlled sufentanil concentration and the end tidal sevoflurane concentration. Our results also showed patients’ HR could be depressed to some degree with the increase of sufentanil plasma target concentration (Table 3). However, the decrease in HR did not result in a decrease of patients’ MAP, especially when a high concentration of sufentanil was administrated. It implies the hemodynamic safety range of sufentanil is large, which is consistent with the results of Fechner and his colleagues [31]. Our study did show that the use of sufentanil at a large dose results in a delay of anaesthesia recovery (Table 1). Therefore, the administration of larger dose sufentanil for short surgery such as laparoscopic cholecystectomy is not recommended.

There are several potential limitations to our study. First, we did not measure arterial blood gases during the pneumoperitoneum period. Although the end-expiratory CO_2_ partial pressure was maintained in the normal range by adjusting the ventilator, we did not measure the actual CO_2_ partial pressure to exclude the influence of hypercarbia on the sympathetic adrenergic response. Second, we did not measure the actual plasma sufentanil concentration. Although the Bovill pharmacokinetic model for target-controlled infusion has been shown to be safe in Asian people, it would have been desirable to measure the actual plasma sufentanil concentration to exclude individual error. Third, we did not monitor muscle relaxation. The level of neuromuscular blockade may influence the relaxation of the abdominal muscles, so as to affect the ease of creation of the pneumoperitoneum and thereby affect the adrenergic response during CO_2_ insufflation.

## Conclusions

The MAC_BAR_ of sevoflurane can be decreased with increasing sufentanil plasma target concentrations. A ceiling effect of the decrease occurred at a sufentanil plasma target concentration of 0.5 ng ml^− 1^. When the sympathetic adrenergic response was inhibited in half patients to pneumoperitoneum stimulation in each group, the changes of E and NE concentrations showed no significant differences.

## Data Availability

The datasets used and/or analyzed during the current study are available from the corresponding author on reasonable request.

## Abbreviations

MAC_BAR_: Minimum alveolar concentration of sevoflurane for blocking adrenergic response
E: Epinephrine
NE: Norepinephrine
CI: Confidence interval
BMI: Body mass index
ASA: American Society of Anesthesiologists
CO_2_: Carbon dioxide
BIS: Bispectral index
MAP: Mean arterial pressure
HR: Heart rate
PACU: Post-anesthesia care unit
