# Correction to: Effects of different sufentanil target concentrations on the MAC_BAR_ of sevoflurane in patients with carbon dioxide pneumoperitoneum stimulus

**DOI:** 10.1186/s12871-020-01184-7

**Published:** 2020-10-23

**Authors:** Yanxia Guo, Dan Wang, Xiaolin Yang, Pingping Jiang, Juan Xu, Guoyuan Zhang

**Affiliations:** 1 Department of Anaesthesiology, Affiliated Hospital of North Sichuan Medical College, Nanchong, 637000 Sichuan China; 2 Department of Clinical Laboratory, Affiliated Hospital of North Sichuan Medical College, Nanchong, 637000 Sichuan China

**Correction to: BMC Anesthesiology 20, 239 (2020)**

**https://doi.org/10.1186/s12871-020-01160-1**

Following publication of the original article [1], the authors reported an error in Fig. 1. The correct version of Fig. 1 is shown below.

**Fig. 1:**
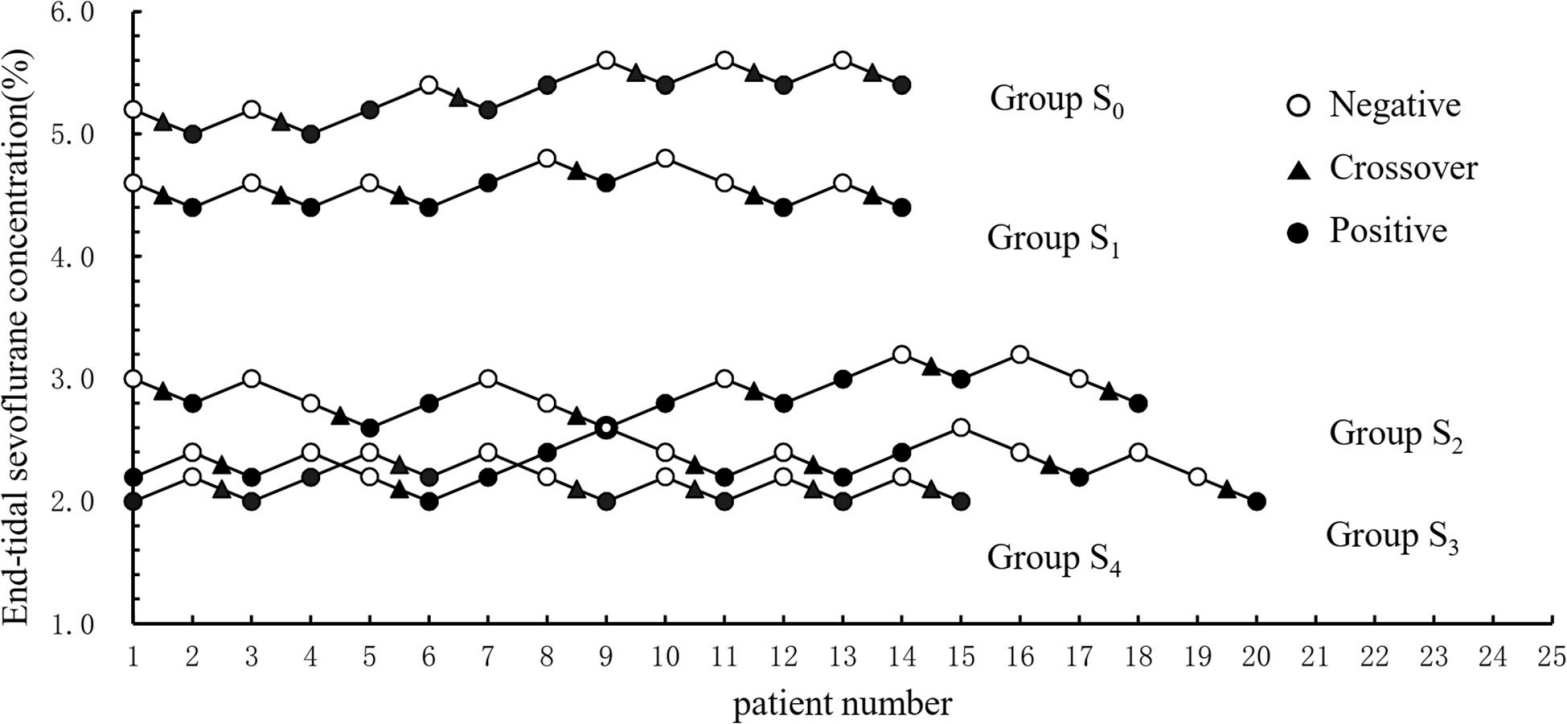
Dixon up-and-down plots for each group. The plasma target concentration of sufentanil in groups S_0_, S_1_, S_2_, S_3_ and S_4_ was 0.0, 0.1, 0.3, 0.5 and 0.7 ng ml^− 1^, respectively. The empty (solid) circle represents the negative (positive) reaction to hemodynamics parameters, and the triangle indicates the intersection of negative and positive reactions. The ninth patient was given the same concentration of sevoflurane both in group S_2_ and group S_3_. To get six crossovers, 14, 14, 18, 20 and 15 patients were needed in groups S_0_-S_4_, respectively
